# Rett Syndrome. Guidelines for Individual Intervention

**DOI:** 10.1100/tsw.2006.252

**Published:** 2006-12-06

**Authors:** Meir Lotan

**Affiliations:** ^1^ Israeli Rett Syndrome Association, National Rett Syndrome Clinic, Chaim Sheba Medical Center, Ramat-Gan, Israel; ^2^ Zvi Quittman Residential Centers, Israel Elwyn, Jerusalem, Israel; ^3^ Department of Physical Therapy, Academic College of Judea and Samaria, Ariel, Israel

**Keywords:** Rett syndrome, guidelines, individual intervention, therapy, Israel

## Abstract

Rett syndrome (RS) is a neurological disorder affecting mainly females. RS is considered the second most frequent cause for severe and complex neurological dysfunction in females after Down syndrome. Patients with RS are characterized by an array of neurological and orthopedic difficulties that mandate an intensive therapeutic intervention program for the duration of the individual's life. Many aspects of the clients well-being and functional status depend on the therapeutic intervention she receives and on her compliance to it. This article will briefly review common intervention approaches for individuals with RS and their present day's application. Due to the notion that individual intervention is the foundation on which progress and development of the functional gains rests, the present article will place basic guidelines for individual intervention with clients with RS. The article is mainly based on the clinical experience of the author and others working with individuals with RS.

## INTRODUCTION

Rett syndrome (RS) is a neurological disorder expressed mainly in females[1,2], characterized by normal birth and apparently normal psychomotor development during the first 6–18 months of life[3]. The child then enters a short period of developmental stagnation, followed by rapid regression in language and motor skills. The trademark of the disease is the repetitive stereotypical hand movements appearing when the child enters stage II of RS. Additional characteristics at the breakthrough of the disorder include autistic like behavior, panic-like attacks, bruxism, breathing irregularities, episodic of apnea and/or hyperpnea, gait ataxia and apraxia, tremors, and acquired microcephaly[4]. After this period of rapid deterioration, despite the relatively stable manner of the disease, the child with RS is likely to develop dystonia and if not ambulant, might also develop foot and hand deformities[5] as they grow older. Seizures occur in 85–50%[3,6 correspondingly] of individuals with RS. Females with RS typically survive into adulthood and their estimated life expectancy is about 50 years[7].

The difficulties presented by the individual with RS and, on the other hand, their longevity, necessitate individual adaptation of intervention in all educational and therapeutic areas. Research shows that brain development is continuing after the onset of RS[8] and that cognitive and communicative abilities of the individual with RS is not deteriorating across the years; hence, setting proper, achievable therapeutic educational and communicational goals for the individual with RS is vital[9]. Since overcoming all the above-mentioned impediments require a therapeutic intervention for many years, it is imperative that such treatments will be delivered to the individual with RS and her family with outmost proficiency. Therefore, treatment planning should be related to assessment of the individual's current levels of functioning as well as her strengths and weaknesses, yet this basic primary component will be left outside the scope of the present article.

## GENERAL COMMENTS REGARDING THERAPEUTIC INTERVENTION

A growing body of research recommends that intensive, sustained treatment is important in order to improve the long-term outcome in syndromes under the umbrella term of PDD (pervasive developmental disorders)[10]. Intervention planning should, on one hand, be grounded in the present functional and medical reality, but on the other hand, should also focus on the long-term vision\prognosis for the client's potential. Planning entails attention to educational interventions, residential situations, and vocational programs[11,12,13]. A well-functioning intervention program must also include appropriate involvement and collaboration with the individual's family[14]. Intervention should be focused and individualized, and must be broadly implemented to relate to the full range of impairments shown by the client. Regardless of the individual's age, treatment planning should include provision for structured opportunities for learning and for generalization of what is learned. Individuals with RS require high-level general medical and paraprofessional care, which includes provision for routine preventive health measures as well as for the special needs of the multihandicapped person.

Since many disciplines are involved in different aspects of the treatment, due to the diverse phenotypic expression among clients with RS and over the course of the disorder, it is important that one clinician be primarily involved with the parents to develop a plan of care for the child. This individual must also be responsible for service coordination and advocacy[10]. Clinicians should help to coordinate services and work with parents to obtain appropriate educational programs, advocate for services such as respite care, provide support for the family, and provide consultation regarding prognosis of the disorder as well as the therapeutic, medical and pharmacological management[13].

## CREATING A THERAPEUTIC NET AROUND THE CLIENT

Educational services are a central and integral aspect of the treatment of children and adolescents with RS. In most western countries, the law mandates the provision of an appropriate educational plan for all children including those with special needs. As part of this educational program, additional services, such as speech/language therapy, occupational therapy, physical therapy, and others, are often required. While early intervention undoubtedly is very helpful[15,16], it should be administered while gathering information and addressing issues such as: What features of the treatment are most important and to whom? Should those issues be enhanced? What characteristics of the child are associated with greatest improvement? Should the management program focus on such issues or address all difficulties? What are the present and future targets goals for the client?

It is agreed that for this population, sustained and continuous programming is more effective than episodic programming (meaning that the option for summer programming should be positively considered because children under the umbrella tem PDD might regress in the absence of appropriate services)[10].

## Supplementary Therapeutic Services

Stretching a therapeutic net around the client with RS means that a group of therapeutic approaches are needed when aiming at fully supporting the individual and her family's needs. Such therapeutic agents might be conventional or unconventional, but it is advised that they collaborate to support jointly the full-scale needs of the individual with RS. A partial list of intervention approaches are:

- **Special education (SE)** — The concept of "least restrictive environment" implies the integration of students with disabilities in mainstream school programs. The importance of the educational environment lies in the fact that school represents many academic and social learning opportunities, beyond traditional classroom instruction. Students who are integrated in to the mainstream educational environment are exposed to those opportunities. All students require support from teachers, classmates, family, and friends in order to thrive and to gain full benefit from their school experience. Students with RS have special needs that require additional supports beyond those ordinarily received in the school setting. Students with RS must have access to an education that will enable them to develop the knowledge and skills they need in order to participate in their surrounding society to their fullest ability. The SE teacher is the heart of the educational system in charge of implementing the theoretical concepts into an actual achievable daily curriculum. The SE role is to mediate between the child's educational and family surroundings, and to deliver the educational plan to her class. Furthermore, the SE teacher should integrate all intervention programs for pupils with RS through the construction of the IEP (Individual Educational Plan) and should supervise its execution throughout the academic year by interweaving it into the pupil's daily activities[17].
- **Physical therapy (PT)** — Individuals with RS are struggling to overcome a vast array of orthopedic and neurological difficulties. Coping with such difficulties and overcoming the associated limitations is a wearisome task for the individual with Rett and for her family. The therapist can enhance the child's physical fitness and function[18], thus reducing the secondary dangers of immobility. The PT professional can also design and implement a functional intervention plan applied by the therapist him/herself or by others. At the educational surrounding, the therapist is responsible for integrating his/her program into the child's daily educational curriculum by means of the IEP. An appropriate and intensely applied PT regime can help the child and her family to cope and even overcome some of the above-mentioned limitations. The therapist should advise other team members regarding issues within his/her field of expertise. Joint intervention sessions with other professions are highly recommended[19], as well as the use of supplementary environments such as the Snoezelen[20,21].
- **Music therapy (MT)** — Andreas Rett (1924–1997) recognized the potential of music to penetrate the heavy shield of disability that masks potentially hidden abilities of individuals with RS[22]. Music therapists use their applied musical skills of improvisation and songs to establish rapport with the child, utilizing it as bridge to bond and engage in nonverbal forms of communication. MT can open channels that, through musical sounds, can offer a child new opportunities. With support, the individual with RS can express emotions and feelings, further enabling some communication and learning to take place. Current knowledge on the use of MT as an intervention for the individual with RS suggests that MT can enhance functional hand use and reduce stereotypic hand movements, develop possibilities for choice making, enhance vocalization, improve attention and eye contact, and develop emotional-communicative channels that can either facilitate the individual with RS or, on the other hand, relax and sooth her[23,24,25,26,27,28,29,30,31].
- **Speech therapy (ST)** — Most individuals with RS have difficulties with expressive language (talking) and it is believed that their ability to internalize (understand) incoming information is far greatly attained than their ability to express themselves verbally. The ST professional can increase expressive communication of the individual with RS by using different Assistive

Augmentative Communication (AAC) devices. Eating is a field that speech therapists are highly trained in, and since most individuals with RS present some forms of difficulty in this area[32], the therapist can help the person with RS to overcome apraxia and other basic motor difficulties that are involved with the complex task of eating. It is advised that the speech therapist work jointly with other therapists, staff, and family to explore all potentially successful means of communication, and to use effective positioning so that the individual may be fully free to reach her utmost abilities.

- **Occupational therapy (OT)** — Individuals with RS shows difficulties in areas of manual activities, sensorial perception and processing, and also in activities of daily living (ADL), such as eating, self-care, and toileting. Occupational therapists are trained to make the unachievable task attainable. The OT professional provides ways to help the individual with RS to increase the use of her hands and body by adapting the client's environment. Enhancing the individual's function will improve her satisfaction and confidence and presents her with an improved ability to control her own world. The lack of sensory mastery by the nervous system of the individual with RS leaves her with inability to interpret the world around her correctly. The therapist can help the client with RS to better organize the information she receives by using various therapeutic approaches and techniques, such as sensory integration (SI), brushing, swinging, tactile activities, and deep tissue compression. As one of the team members embracing the individual with RS, the OT professional can recommend adjustments of the client's surroundings in many daily situations to other team members.
- **Hydrotherapy (HT)** — Hydrotherapy presents the pleasant engulfment in warm water while at the same time is helpful in reducing spasticity and in softening rigid tissues. The HT professional can use the water to provide the individual with RS with intense stimulation, thus enabling a better sensory input. The use of warm water might calm the individual with RS who is afraid of motion. The person with RS is able to move easily and freely in the water, without fear of falls. Furthermore, spending time in a pool places the individual with RS at a normative place where she can associate with peers and family. Other advantages suggested by hydrotherapy are the pleasure of new and exciting experiences through water activities and the ability to function in ways she might not be able to achieve on land. In some cases, the water enables the individual with RS to express her lost and sometimes latent motor skills[33].
- **Pharmacological and related interventions** — Although not curative, medications may be useful for relieving symptoms that interfere with participation in interventions[34], or alleviate sources of impairment or distress experienced by the individual with RS (constipation, seizures, osteoporosis, etc.).
- **Alternative treatments** — Given the chronic nature of RS and the lack of knowledge regarding a cure, it is not surprising that putative cures surface quite frequently, many without substantive background. Not uncommonly, initial reports relate to an individual client or a handful of cases. Such reports are difficult to interpret as the many aspects of the presented intervention approach might be vague or highly uncertain. Such reports usually are not followed by more rigorous, controlled studies in the peer-reviewed scientific literature. Families should be helped to make informed decisions about their use of alternative treatments. Treatments that pose some risk to the individual and her family should be actively discouraged.
- **Follow-up assessments and ongoing treatment** — Due to the fluctuating nature of RS, therapeutic services must be flexible and possess the ability to change at different points during the client's development (e.g., due to increase in antiepileptic medication, the individual with RS who could walk independently a week ago might now find it hard to stand). To provide the specific care needed for this population, coordination of services and family support is an imperative aspect of ongoing care. The nature and intensity of therapeutic contacts depend on the clinical situation and needs of the individual, and should be constantly monitored and adjusted. In light of all the above theoretical background, it would be appropriate at this point to evaluate the present state of therapeutic intervention actually presented for the individual with RS.

## THE STATE OF THINGS — THERAPEUTIC INTERVENTIONS FOR RS TODAY

The present state of therapeutic interventions for individuals with RS was investigated by Leonard et al.[35]. Their inquiry entailed 86 families of individuals with RS, with various ages from infancy to adulthood, and different countries around the globe. Their findings suggest that individuals with RS receive an array of therapeutic interventions according to the following specification.

**TABLE 1**
**Therapeutic Interventions for Individuals with RS**

| OT | PT | ST | HT | Hippotherapy | MT |
|---|---|---|---|---|---|
| 84.9% | 77.9% | 76.7% | 29.1% | 17.4% | 9.3% |

The full scale of these findings was discussed in the original article, but some points should be emphasized. The findings suggested that 36% of parents were dissatisfied with the intervention suggested for their daughters due to insufficiency of intervention, or inappropriateness to RS. Music therapy which has been repeatedly described as of utmost value to this population[22,28,29,30,36,37] is only available for less then 10% of individuals with RS.

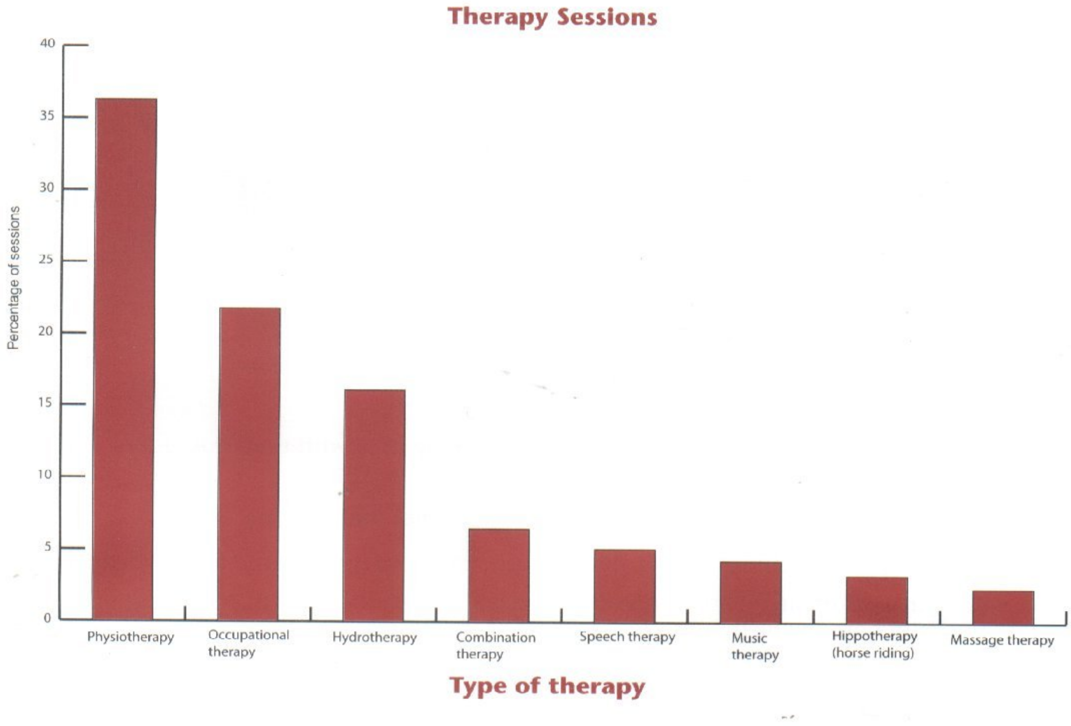

**FIGURE 1.** Therapeutic intervention for individuals with RS. Published with permission from Leonard[38].

Possible conclusions that might be drawn from the article of Leonard et al.[35] is that despite the high functional dependency presented by most individuals with RS (as compared to individuals with Down syndrome), intervention is not administered to all. In most cases, the intervention is not perceived by the medical milieu under a rehabilitative concept and the intervention delivered is often insufficient and inappropriate. The data presented by Leonard et al.[35] revealed that intervention services were insufficient to a complex condition such as RS and should be increased by active efforts of caregivers and parents.

In light of the findings by Leonard et al.[35], the next section will suggest insights into intervention for individuals with RS. We encourage therapists to apply multidisciplinary models of intervention for individuals with RS[39], whether it is delivered as individual intervention[40,41], or in other forms[19], but with attention to the special features of RS.

## INSIGHTS TO INDIVIDUAL INTERVENTION

It is important to reiterate that individual intervention is not perceived as the ultimate intervention method, but merely one basic step to support integration and independence of the individual. The individual intervention is the foundation on which progress is based, especially for individuals with RS, or as Abraham Harold Maslow (1908–1970) said: the “ordinary (individual) therapy may be conceived as a miniature ideal society of two”[42, p. 263]. Due to this concept, the present article submits guidelines for individual intervention with the client with RS.

Adopting the suggested guidelines will help to advance the individual with RS in her struggle to overcome all the debilitating challenges put fourth by RS. The one-on-one intervention may start at the therapy room, but must simultaneously develop in the classroom, the residential environment of the child, and to her peer group and community. At first encounters, the individual with RS might present difficulties typical for all new students. Such times have been reported in the past as times of anxiety for the child and parents as one[43].

Therefore, despite the fact that ultimate performance in the natural environment will not be achieved in the therapy room, it is recommended that initial contact between the client with RS and her therapist be conducted in a familiar quiet place, with a caregiver/family member that the child is familiar with and trusts. In such a favorable environment, bonding between the individual with RS and her therapist will create the necessary milieu for later functional advancements. Achieving rapport during the few first encounters is not always easy and even after the establishment of such a connection, the advancement of the intervention with the individual with RS is slow and demanding, requiring much patience by the therapist.

The connection between client and therapist is sometimes influenced by the emotions of the therapist working with the individual with RS[44]. In new and unfamiliar situations, or when the therapist is tired, depressed, or nervous, results are seldom achieved[44]. If the therapist is attentive to him/herself and to the individual with RS, therapeutic results are much more easily gained. A deeper acquaintance with the person's daily program and her physical and human environment will enable improved communication and meaningful attachment, and therefore is highly recommended. Such bonding will enable the therapist to better decipher the client's signs and his/her reactions to her signals will improve. As a result, the individual with RS's communicative "output" might improve, helping to overcome barriers in education and personal contacts[43,45,46].

Despite the fact that it is probably a well-known feature to any therapist/educator, the author would like to reiterate that "individual intervention" does not mean that every therapist is totally devoted to his private therapeutic agenda and completely oblivious to other team members. Any therapist/educator working with an individual with RS must maintain professional affiliations with the client's parents and additional paraprofessionals contributing to her curriculum. It is advised that all team members (working according to a multi/transdisciplinary approach) join up and decide together on mutual goals for the client with RS, grouping their efforts to advance the individual with RS according to the mutually decided intervention plan. In some cases, parents will seek additional treatments outside the educational setting and it is important that providers of such services coordinate their work with that of the educational facility[10].

Intervention goals are built in accordance to each client's abilities and needs, and are lead by the joint intention of achieving the highest functional level and the best quality of life attainable. Lewis and Wilson[43] have mentioned the obvious, yet important statement, that individuals with RS are complete human beings and are not assembled by sites in need of education, and\or areas requiring improved mobility, and\or locations seeking medical attention. Therefore, the intervention with the individual with RS should be holistic, integrating all aspects towards the achievement of maximal gains[43]. All therapeutic interventions should address difficulties in performance by the individual in daily activities (eating, drinking, communicating, standing, walking, communicating, and socializing).

The recommendations suggested thus far might be agreed on for many individuals with complex disabilities. The next section will specifically address insights in to intervention with RS and represents the gatherings of educational/therapeutic experience collected over the years by professionals working with this population.

## INSIGHTS FOR INDIVIDUAL INTERVENTION

The aim of this last part of the article is to establish a body of knowledge that will help the clinician to approach the client with RS. Despite the fact that every individual with RS is a unique entity, different from others with RS, and regardless of the obvious knowledge that each treatment is the consequence of a thoroughly delivered evaluation, there are similarities among individuals with RS, which the following guidelines take in to account.

1. The cognitive ability of individuals with RS is unclear. Conventional intelligence quotation (IQ) tests mainly rely on manual ability or on oral expression; hence, addressing the weakest abilities of this population. Until appropriate tests are administered, the therapist should believe in the ability of individuals with RS and work with them with respect and with the assumption that they perceive much more than their ability to produce.
2. In order for the intervention process to achieve maximal results, the paraprofessional is advised to achieve the trust and confidence of the client with RS; therefore, it would be wise for the therapist/educator to invest in achieving a strong rapport before and during intervention implementation. Such a connection may later prevent rejection and discomfort[47].
3. It would greatly benefit the individual with RS if all caregivers (siblings, parents, therapists, and educational staff) could be involved or at least familiarized with the intervention program. Systematic meetings are recommended to enable information transfer (data regarding the individual’s natural communication acts, her preferences in colors, food, drinking, activities, and more) from the educational arena to the client’s residence and vice versa. Keeping constant open communication paths between meetings is advised.
4. When handling the individual with RS, it is of utmost importance to understand her world. Most individuals with RS can open up to the therapist, using their developed emotional capacities. If the therapist/educator will stay attentive enough, he/she will be able to detect the person with RS signals.
5. In order to promote a sense of safeness by the individual with RS, it is preferable to maintain quiet familiar surroundings, and to employ consistency in applying the intervention program.
6. Presenting the individual with RS with proper motivational factors could greatly enhance her cooperation, thereby upgrading her functional abilities (i.e., an individual with RS that was given physical therapy sessions by the author, usually showing typical poor hand function, had the ability to eat a sandwich with chocolate spread or drink a whole glass of Coca-cola, but only these specific food items, holding both the sandwich and the glass firmly with both hands, for the duration of finishing her meal).

7. It is for the best if the individual with RS considers her world to be safe and comprehendible before attempting to participate actively in it. Therefore it is advised to:
   · Apply therapy sessions at preset times
   · Explain future happenings in advance
   · Give the individual ample time to accustom herself to the situation (the room, equipment, therapist)
   · Construct each session from well-acquainted sections
   · Support the individual when she seems baffled\disoriented
   · Announce the end of therapy in advance
8. If the individual with RS is extremely attached to a person\object that supplies her with confidence (a caregiver, pacifier, a corner in the room), the therapist should consider using that as a safe starting point when new demands or exercises are presented.
9. Individuals with RS love music and songs[48]. They react much better to songs than to a direct spoken instruction. They enjoy whispers in their ears and different voice games, and prefer live singing over instrumental music. Using music during therapy could extremely motivate the person with RS to participate actively in individual intervention[49]. If the person with RS does not seem enthusiastic from the music played for her, please bear in mind that each individual with RS presents a personal flavor in music not necessarily compatible with the therapist’s taste.
10. Most individuals with RS do not communicate verbally, but this does not mean they have nothing to say. Since body language and eye gaze are silent ways of communicating, it is the responsibility of the therapist to be alert and attentive to the client’s signs. If it is known that a specific individual with RS has some distinct gestures with specific meaning, such knowledge should accompany the individual throughout her daily encounters with peers and adults. When the individual with RS is using such a gesture, proper acknowledgment is due even if the person’s needs cannot be answered at that specific time. (For example, the individual with RS is signaling she would like to watch a video film during lunch or a family trip – "Yes, I can see that you want to watch a video, but we can't do it right now; we can watch a video film when we get home\to class\finish eating.")
11. Most individuals with RS can communicate through facial expression. The attentive therapist/caregiver will be able to react to their fear, curiosity, anxiety, enjoyment, anticipation rejection, and much more.
12. Individuals with RS might sometimes present vocal abilities after they are acquainted with their surroundings and have accepted the therapist. React with your own voice and initiate a mutual conversation. When the individual with RS will feel that her human environment is responding to her messages, she will increase such outputs.
13. Routine is important for the individual with RS. It is advised to supply her with constant familiar rituals matched with verbal or visual symbols. (For example, before food is served, you sit around the table, talk about food, put plates and eating utensils on the table, and look at pictures of different food items that will be presented shortly; before going to the swimming pool, you get undressed, wear your bathing suit, look at a picture of the pool, and talk about doing hydrotherapy….)
14. Routinely addressing the indivudual daily curriculum will make her feel safe and confident, thus encouraging her to express her abilities fully. If a change is planned in her regular activities, it would be for the best to inform the individual with RS and try to make the change as gradual as possible for her to adjust.
15. The individual with RS finds it difficult to generalize due to her apraxia. (For instance, an individual that will put her hand on the caregiver's arm, at lunch time, to sign for more food will not use such a gesture to sign for the need to go to the bathroom.) It is recommended that an existing gesture is used to generalize a communicative act from one location to other situations.
16. When implementing a program for an individual with RS make sure it is:

- Clear to the client as well as to yourself (what do you want to implement, what is the primary and final goals)
- Structured (the method, the location, other participants, appliances)
- Entailing opening and closing rituals
- Keeping the individual interested and involved during all part of the program
- Maintaining and supporting the client's level of confidence
- Changing (if change is due) gradually

17. When constructing a therapeutic intervention program, it is recommended that the program be as comprehensive as possible, entailing the home as well as the educational/rehabilitation facility. (The home as a support unit for the therapeutic program — as much as it is important for the parents to participate fully in the individual's intervention program, they can only do so in accordance with their emotional state. The therapist should bear in mind that they are first and foremost the child’s parents responsible for mainly offering love and care.) It is my experience that without such wide-range support, most programs might bear negligible results or just fade in time.
18. The recommended duration of therapeutic sessions acceptable for most individuals with RS is around 20–30 min. Nevertheless, the time table of therapeutic interactions should not be a-priory reduced, but rather depend on the capability of the individual with RS. When music and warm loving care are intertwined within the therapeutic interaction, the individual with RS can sometimes be kept attentive for hours. When she decides she has had enough, she will show the therapist in her specific way that the session has come to its end (closing her eyes, rolling her eyes backwards, falling asleep, losing interest…etc.).
19. Individuals with RS are capable of learning[49]. Therefore, decide on the skill you want the individual with RS to acquire and repeat the chosen activity for the duration of time needed for her to internalize this new skill.
20. Due to apraxia, the individual with RS will find it difficult to learn new skills and to perform motor acts[50]. Continuous repetitions are advised to reduce the individual's need to organize and plan her performance, thus reducing her reaction time by making the execution of a motor act automatically performed. When results are slow to emerge, in some occasions, patience and perseverance might be the only courses of action. The apraxic individual sometimes requires a few months to handle a new task.
21. On the other hand, I have witnessed situations where the therapist was asking the individual with RS to perform an act repeatedly (e.g., touch pictures or objects named over and over) until the child revolted by suddenly refusing to cooperate. In such a situation, the therapist\educator should be assured that the client did not suddenly forget everything she previously knew. I believe such situations could be avoided if interactions are planned in a more versatile and challenging way, and the therapist gives acceptance and belief in the client’s ability.
22. Daily activities are best examples for spontaneous learning experiences for the individual with RS. (Eating is a chance to talk about farming and food making; bathing is a chance to develop body schema and reduce sensorial sensitivity by applying massage.)
23. Individuals with RS might show some difficulties in regards to sequence acquisition. In such cases, it is recommended that the learning program be adjusted for the learner by breaking it to simple steps.
24. It is recommended that general remarks (such as, "go up the stairs") are used instead of direct, specific instructions that demand exact motoric planning (such as, "put your hand on the railing"). Using more general forms of speech might speed up the individual reaction.
25. When instructing the individual with RS, it is recommended that the therapist guide her by using the same instructions. The therapist is best to refrain from changing the way he\she is phrasing their guidance, since it might confuse the client, distracting her from achieving her goal.
26. Due to the long reaction time of individuals with RS, the therapist is advised to wait patiently for the client to perform following instructions (during that time the individual with RS might appear distracted, will accelerate her breathing pattern, look to the sides, and perform intense body rocking and hand stereotypical movements). All these behaviors are just her way of preparing her reaction. Repeating the former request during that time will cause her to reset her intention, restart planning her reaction, causing her to further elongate her reaction time, or even neglect her desire to participate.

27. One of the strengths of people with RS is their visual system. The use of pictures should be extremely encouraged[47] as communication and educational channels, as means to enable choice in daily situations, and also as a motivational factor (e.g., when using pictures of familiar characters). The constant use of pictures is most empowering for the individual with RS.
28. Integrating different aspects of one experience is highly recommended and is of great importance. (For instance, how can we expect the individual with RS to associate between a picture of a big red round apple with those half-moon-shaped white slices on her plate, if she has never seen an apple sliced into pieces?).
29. Some individuals with RS are so handicapped by the combination of ataxia, apraxia, distonic muscles, and long reaction time that performing an accurate manual task might be impossible. For these individuals, any initiation and attunement towards the requested goal should be praised, even if the action itself was "unsuccessful" (such as, the individual was asked to get a doll off the table and instead struck it to the ground), so she will not be discouraged from trying again.
30. On the other hand, automatically and intensively congratulating an individual with RS for anything she does might cause the praise to become gradually redundant.
31. It is important to note that the sleeping patterns of the individual with RS are sometimes immature (waking very early in the morning, disrupted sleep sequence[51]), causing the individual to drowse off during daytime. The use of anticonvulsive medications might also cause the individual to become drowsy and slow to react. It is advised that such circumstances be taken into account a-priory when planning the individual intervention for the person with RS, inserting such interactions during periods where she is usually known to be at her functional peak.
32. Individuals with RS are famous for their functional fluctuations. During moments in an activity (but sometimes even for hours or days), the individual with RS may seem lost and disconnected; “It is as if her mind is covered by clouds"[47, p.77]. In some cases in the past, such a behavior was found to reduce the staff (caregivers and therapist alike) confidence in the client’s abilities. One must not give up on the client with RS! This phenomenon is caused by numerous factors associated with this disorder and the therapist should have confidence in the fact that the individual with RS will reconnect and return to her previous functional abilities.
33. Most individuals with RS will find it very hard to split their attention between several issues. If we are trying to work on one of our therapeutic\educational goals while the individual is trying to convey a different message, she will not pay any attention to story telling, computer games, or whatever task we had in mind. In such occasions, it is better to take time out, address her topic, and then it might be possible to return to the former planned task.
34. In some cases, the individual with RS could be in the “wrong place” (such as, she is sitting in the kitchen and wants to see a video movie in the living room). Her wishes in such circumstances will be difficult to understand. If the individual with RS’s wishes are unclear, try to raise many options and do a lot of trial and error tests (using your questions and the individual response) until her wishes become clear. Such episodes (if successful) are the best communication enhancers.
35. When assisting the individual with RS in performing a task, timing is everything. If we try to hurry her performance, she will probably react by clenching and resisting our “push”. It is most helpful and productive if the caregiver\therapist\educator is familiar with the client’s rhythm, and is attentive to the individual’s movements, presenting his\her assistance at the right time, direction, force, and pace.
36. The siblings of the individual with RS are usually her natural therapists. They share remarkable bonding with one another, and the therapist might consider incorporating them into the therapeutic sessions or at least learn from the way they handle themselves with their sister.

37. Age-appropriate intervention should be advocated, as well as enabling the individual with RS to participate in peer activities.
38. Various difficulties impede the individual with RS from achieving independent function. It is advisable that an "independence intervention" be created at regular points in time and settings. In these situations, the individual with RS will set the rules of the interaction. This form of intervention will empower the individual, encourage her to initiate, and expose her true potential.

## SUMMARY

The individual with RS is at many times extremely debilitated and handicapped by the vast array of neurological, orthopedic, and functional limitations posed by the Rett disorder. Since the aim of the therapist is to improve the client's quality of life by overcoming or reducing these limitations, an individually tailored intervention program should be implemented.

Such a program should be the result of a well-performed evaluation, holistically employed by a group of caregivers and therapists. The intervention program originating from such an evaluation should create a continuous network of human support around the individual with RS.

It is clear that some of the insights in the above-mentioned guidelines might contradict one another. Such contradictions could only be avoided if the therapist is attentive to functional and emotional alterations, and applies a flexible therapeutic regime that constantly adapts to the changes presented by the client with RS. It is the belief of this author that individuals with RS exceed many of their limitations if such a comprehensive, individually tailored, and well-suited therapy program could be implemented.
